# Duodenal Obstruction during Pregnancy

**DOI:** 10.1155/2022/3516542

**Published:** 2022-02-09

**Authors:** Mansooreh Haghiri, Sedigheh Borna, Kamran Hessami, Ali Sharifi, Seyed Mohsen Ahmadi Tafti, Mahrooz Malek, Nasim Pourdamghan, Sedigheh Hantoushzadeh, Abolfazl Shirdel Abdolmaleki, Maasoumeh Saleh

**Affiliations:** ^1^Maternal, Fetal and Neonatal Research Center, Vali-e-Asr Hospital, Tehran University of Medical Sciences, Tehran, Iran; ^2^Department of Obstetrics and Gynecology, Baylor College of Medicine, Houston, TX, USA; ^3^Maternal-Fetal Medicine Research Center, Shiraz University of Medical Sciences, Shiraz, Iran; ^4^Department of Surgery, Beesat Hospital, Hamadan University of Medical Sciences, Tehran, Iran; ^5^Department of Surgery, Tehran University of Medical Sciences, Tehran, Iran; ^6^Department of Radiology, Vali-e-Asr Hospital, Tehran University of Medical Sciences, Tehran, Iran; ^7^Department of Obstetrics and Gynecology, Kerman University of Medical Sciences, Tehran, Iran, Kerman, Iran; ^8^School of Medicine, Shiraz University of Medical Sciences, Shiraz, Iran

## Abstract

Intractable vomiting and elevated liver enzymes during pregnancy seem to be associated to the obstetric etiologies; however, other causes such as acute surgical emergencies should be considered. The patient was a 26-year-old woman at 18 weeks of gestation with intractable vomiting, intolerability of oral intake, weight loss, and absence of abdominal pain. Her physical examinations and laboratory tests had no remarkable findings except elevated liver function test (LFT) and hypokalemia. Considering the lab data and normal abdominopelvic ultrasound, magnetic resonance imaging was performed which revealed dilation of the D1-3 and collapse the D4 sections of duodenum. She underwent exploratory laparotomy which confirmed duodenal obstruction caused by Ladd's band. After the Ladd's operation, the patient started oral intake of nutritious, and her LFT decreased to normal ranges. After the last follow-up, she has had gained 18 kg and gave birth at 36 weeks of gestation due to the premature rapture of membranes and delivered a 2 kg small for gestational age otherwise healthy infant. The experience gained from this case was to consider all possibilities (such as small bowel obstruction) and evaluate them in a pregnant patient to consider other causes of nausea, vomiting, and abnormal LFTs in a pregnant patient.

## 1. Background

Acute intestinal obstruction during pregnancy has been described with different incidences such as 1/66,431 and 1/1500 [1]. This condition is very important and emergent during pregnancy which requires proper treatment as soon as possible as same as other abdominal emergencies [1–3]. The most common causes of mechanical obstruction in pregnancy have been reported as adhesions, volvulus, and intussusception with the prevalence of 58%, 24%, and 5%, respectively [1]. So far, some reports have highlighted malrotation as an uncommon cause of small bowel obstruction (SBO) during pregnancy [4–7]. Congenital malrotations are mostly asymptomatic in adults and have been known as a rare cause of SBO in this age group. Despite the different etiologies of SBO during pregnancy, the most common symptoms are abdominal pain (98%), vomiting (82%), and tenderness (71%) in the abdomen. Thus, abdominal pain seems to have a notable sensitivity for SBO in these cases [1].

Herein, we report a very rare case of SBO during pregnancy with persistent progressive nausea and vomiting due to duodenal obstruction caused by a previously asymptomatic congenital condition (incomplete rotation of duodenum). The clinical presentation and laboratory tests of this case were very similar to the other prevalent obstetrics-associated complications. Considering her two previous pregnancies terminated in a similar situation, the current report would bold role of proper imaging modalities and other investigations in the diagnosis and treatment of such cases.

## 2. Case Presentation

A 26-year-old woman (Gravida 3, Para 2, Death 2) was referred to our tertiary care center (*Vali-e-Asr Hospital*) at her 25th week of gestation (WOG) with blood pressure of 100/80 mm Hg (pulse rate 98). She complained of intractable large volume bilious vomiting, generalized weakness, progressive weight loss (13 kg so far), had an episode of bowel movement every 2 to 3 days, and inability to consume food started from 18th WOG (the patients received IV fluid therapy several times as outpatient between 18 and 25 weeks of gestation). Except for two failed pregnancies, she had no remarkable family and psychosocial history. In both of the previous pregnancies, similar presentations to her current condition were found. Those pregnancies were terminated at 26th and 22nd WOG with the diagnoses of acute fatty liver of pregnancy and HELLP syndrome, respectively.

Her first pregnancy (22 years old) had advanced with no complications until the 18th WOG when nausea and vomiting started to be more prevalent and severe as the time passed. Due to the progressive intractable vomiting, dehydration, and hypokalemia, as well as intolerance to oral intake, the patient was completely dependent on intravenous fluid and electrolytes which finally had led to weight loss and severe weakness (in another medical center). During the 25th WOG, liver function test (LFT) values started to rise along with negative laboratory results for viral hepatitis. Also, there were no criteria for autoimmune hepatitis. Since we had no more data regarding this hospitalization, the refeeding syndrome could be a suspect for the impaired LFTs [8]. Finally, the pregnancy was terminated at 26th WOG while suspecting acute fatty liver of pregnancy. Following the termination, she soon tolerated oral intake, and the LTFs returned to the normal levels after a week.

**Figure 1:**
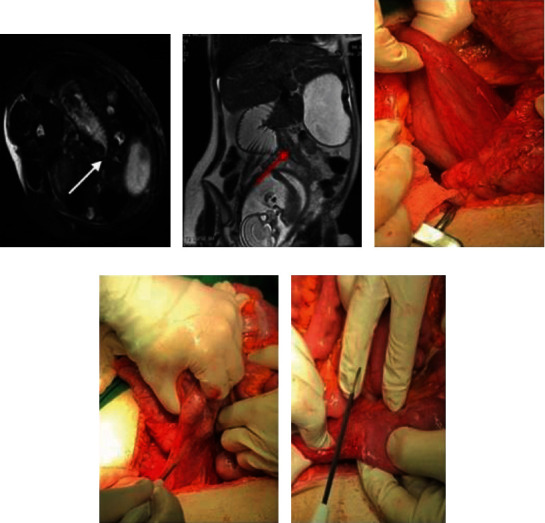
(a) Transverse oblique (patient is lying on a side) view marked dilation of the stomach and duodenum up to D3 level (the arrow is the point of stenosis). (b) The site of Ladd's bands (red arrow), uterus, and duodenum. (c) Dilated duodenum under cecum. (d) Peritoneal Ladd's bands. (e) Released peritoneal band in the site of stenosis.

In her second pregnancy (at the age of 25), similar symptoms to the previous reemerged after the 17th WOG. Intravenous (IV) hydration plus mineral supplement replacement were not able to meet the patient's maintenance needs. Meanwhile, the symptoms became more severe, and LFTs increased with a very quickly and during hospitalization period. The second pregnancy was terminated around 22nd WOG with suspicion of HELLP syndrome. Considering the complications during both pregnancies, the patient was referred to a gastroenterologist for evaluation of Wilson's disease, autoimmune hepatitis, and Crohn's disease. The multidisciplinary workup did not show any remarkable finding of such sort. Before her third pregnancy, she was evaluated by a well-experienced rheumatologist as well, which they did not find any abnormal issue (according to the patient's statement). In the third pregnancy and before referring to our center (around the 22nd WOG), the patient was switched to intravenous nutrition replacement since oral intake was intolerable (by general practitioners because of their suspicion to hyperemesis gravidarum). At the same time and when the patient was under treatment with dexamethasone and full dose of ondansetron with steady clinical status, laboratory results showed a progressive increase in liver enzymes. At the time of admission to our center, the patient's physical examinations, fetal heart rate, and primary laboratory investigations (such as complete blood count, blood sugar, 24-hour urine protein, amylase, lipase, and viral tests) revealed no remarkable finding except for LFTs and potassium levels (Table 1). Also, abdominopelvic ultrasound and Doppler flow of inferior vena cava (IVC), portal, and hepatic veins were normal. As the next step, upper gastrointestinal endoscopy was performed which showed no significant findings such as peptic ulcer or pyloric obstruction. Considering the following results, magnetic resonance imaging (MRI) without contrast revealed marked dilation of the stomach and duodenum up to the third part of duodenum, D3 level as well as collapse in D4 part (Figures 1(a) and 1(b)). Thus, the obstruction site suggested to be between D3 and D4.

After deteriorated clinical status and solid evidence of duodenal obstruction, laparotomy was indicated. The transition zone of duodenal obstruction was observed at the junction of peritoneal bands passing across the third part of the duodenum. At the proximal portion of Ladd's band, D3 was significantly dilated while the distal part, D4, was completely collapsed lying on the right side (Figures 1(c)–1(e)). The peritoneum to the right of the ascending colon was incised, and the anteriorly situated bands were stripped to release the duodenum (Figure 1(e)). After the surgery (Ladd's procedure), the patient remained stable and was admitted to intensive care unit (ICU). After two days, the elevated LFTs were decreased by about 50% compared to the presurgery levels (Table 1). She was discharged from ICU after two days with favorable postoperative recovery and normal oral intake. After being discharged, she was followed up to three weeks after labor. During the follow-ups, she gained 18 kg until the time of delivery (cesarean delivery). She gave birth at the 36th week of gestation as a result of the premature rupture of the membrane. The Apgar score and weight of her neonate were 9/10 and 2 kg (small for gestational age), respectively, and both mother and neonate were discharged after labor with no problem or complaint.

## 3. Discussion and Conclusions

Herein, we reported a rare case of duodenal obstruction during pregnancy caused by Ladd's band. One of the most challenging issues, in this case, was the absence of abdominal pain observed in up 98% of pregnant women with the same condition [1]. Thus, considering the absence of abdominal pain in this patient and presence of more common pathologies with nausea and vomiting (accompanied with elevated LFTs) during the second trimester of pregnancy (such as acute fatty liver of pregnancy and HELLP syndrome), obstruction became a neglected diagnosis in her past pregnancies terminated with other diagnoses. This time, considering all of the already mentioned tests performed for rolling out other possible conditions, MRI was carried out which increased the suspicion of the duodenal obstruction. After the surgery (Ladd's procedure), symptoms and abnormal laboratory tests were also resolved, and results returned to the normal ranges.

According to our knowledge, so far, only two cases of duodenal obstruction caused by malrotation following the Ladd's band pain have been reported. However, there have been different major differences [9, 10]. As Yin et al. have explained, the symptoms of their primigravid case were started at 9th WOG with nausea, vomiting, and intolerance to oral intake. Her clinical symptoms worsen, and serum transaminase levels increased (AST: 100-243 U/L and ALT: 297-536 U/L) which led to hospitalization in the 24th WOG. Similar to the current report, their case declared no abdominal pain accompanied by any of the other symptoms. Following the MRI, they performed laparotomy which confirmed congenital bowel malrotation and Ladd's band. Unlike our case, however, they found no signs of obstruction, bowel ischemia, and volvulus; hence, the Ladd's procedure was not performed for their case. She was just treated with parenteral nutrition and was discharged 6 days after the surgery. Also, their six-month follow-up did not reveal any gastrointestinal (obstetrics not mentioned) issues [9]. Furthermore, Pelikan et al. reported extreme nausea and vomiting in a pregnant woman at 38th WOG with nausea, vomiting, and abdominal pain (unlike our case). Ultrasound and gastroscopy were normal, and in despite the obtained results, all the laboratory tests, including LTFs, were normal. After the labor, an abdominopelvic computed tomography (CT) scan (with IV and oral contrast) was performed for the patient which showed intestinal malrotation due to volvulus. During the laparotomy, they incised the Ladd's bands, and in the ascending colon, the mesenteric veins were found thrombosed. Thus, they performed right colectomy accompanied by ileocolic anastomosis due to the suspicion of the tissue viability [10].

Malrotation of the midgut is an abnormal intestinal rotation and fixation in the embryological development of the gastrointestinal tract. While the majority of such abnormalities remain asymptomatic during adulthood, the symptomatic cases are usually encountered in the clinical setting of duodenal obstruction or midgut volvulus [11]. All and all, this was a case of confirmed duodenal obstruction that commonly goes without abdominal pain but only retching and vomiting which seems to be common findings for this level of obstruction. The most remarkable “takeaway lesson” [12] of this case is not to neglect (any) possible diagnoses in a patient, especially pregnant women with no abdominal pain but progressive vomiting accompanied by impaired LTFs. In such cases, MRI should be used more to reach a correct diagnosis as it has helped before in the rare conditions during pregnancy [13]. Also, it should be noted that during pregnancy (especially second and third trimesters), anatomical and physiological changes could alter asymptomatic conditions to symptomatic issues found to be threatening for both mother and fetus.

## Figures and Tables

**Table 1:** The results of blood laboratory test investigations at admission and postoperative.

Laboratory parameter	Before the surgery	Day 2 (postoperative)	Day *X* (postoperative)
Platelet count	256000/*μ*L	2450000/*μ*L	250000/*μ*L
Aspartate aminotransferase (AST)	329 IU/L	142 IU/L	53 IU/L
Alanine aminotransferase (ALT)	745 IU/L	375 IU/L	12 IU/L
Alkaline phosphatase (ALP)	309 IU/L	240 IU/L	369 IU/L
Albumin	3 gr/dL	—	—
Total bilirubin	1.2 mg/dL	2 mg/dL	1 mg/dL
Conjugated bilirubin	0.4 mg/dL	1 mg/dL	0.2 mg/dL
Prothrombin time (PT)	12.2 seconds	14 seconds	—
INR	1.1	1.1	—
Thyroid stimulating hormone (TSH)	1.2 mIU/L	—	—
Potassium (K)	3 mEq/L	3.6 mEq/L	—
24-hour urine protein	280 mg	—	—

## Data Availability

Any other data regarding this case report would be available at online request from the corresponding author if available.

## Abbreviations

SBO: Small bowel obstruction
WOG: Weeks of gestation
LFT: Liver function test
ICU: Intensive care unit.
